# PET‐measured amyloid beta accumulates at an accelerated rate in Down syndrome compared to neurotypical populations

**DOI:** 10.1002/alz.70357

**Published:** 2025-06-24

**Authors:** Andrew McVea, Alexandra DiFilippo, Max McLachlan, Brecca Betcher, Matthew Zammit, Tobey J. Betthauser, Alexander Converse, Dhanabalan Murali, Charles Stone, Sigan Hartley, Sterling Johnson, Dana L. Tudorascu, Charles M. Laymon, Ann D. Cohen, Davneet Minhas, Weiquan Luo, Chester Mathis, Patrick J. Lao, Beau Ances, Shahid Zaman, Mark Mapstone, Elizabeth Head, Benjamin L. Handen, Bradley T. Christian, ABC‐DS investigators

**Affiliations:** ^1^ Department of Medical Physics University of Wisconsin – Madison School of Public Health Madison Wisconsin USA; ^2^ Waisman Center University of Wisconsin – Madison School of Public Health Madison Wisconsin USA; ^3^ Wisconsin Alzheimer's Disease Research Center University of Wisconsin – Madison School of Medicine and Public Health Madison Wisconsin USA; ^4^ Department of Psychiatry University of Pittsburgh Pittsburgh Pennsylvania USA; ^5^ Department of Neurology Columbia University New York New York USA; ^6^ Department of Neurology Washington University in St. Louis St. Louis Missouri USA; ^7^ Department of Psychiatry University of Cambridge Cambridge UK; ^8^ Department of Neurology University of California Irvine California USA; ^9^ Department of Pathology & Laboratory Medicine and Neurology University of California Irvine California USA

**Keywords:** amyloid beta (Aβ) plaques, Alzheimer's disease, [C‐11]PiB, longitudinal, PET imaging, Trisomy 21

## Abstract

**INTRODUCTION:**

Individuals with Down syndrome (DS) have a high prevalence of Alzheimer's disease (AD) and reveal an earlier age of amyloid beta (Aβ) onset compared to sporadic AD. Differences in amyloid accumulation rates between DS and sporadic AD populations have not been established.

**METHODS:**

Participants with ≥ 3 [C‐11]PiB scans (spanning > 6 years) and transitioning to Aβ+ were included, resulting in 20 DS and 23 neurotypical (NT) participants. Amyloid accumulation was compared using global standardized uptake value ratio (SUVR) for Aβ deposition, with individual growth rates (*r*) estimated using the logistic growth model ($SUVR\;\left(t\right)=SUVR_{BL}+\frac{K}{1+e^{-r(t-t_{50})}}$).

**RESULTS:**

The average growth rate in the DS cohort was 0.28 (0.08)/year versus 0.20 (0.08)/year for NT (p=.002), an increase of 40%.

**DISCUSSION:**

Using individual longitudinal analyses, accelerated amyloid accumulation in DS is observed, This has important considerations for informing treatment trial design and monitoring beta‐amyloid changes in future AD studies involving individuals with DS.

**Highlights:**

- Aβ accumulation rate was estimated using a logistic growth model.
- There was no overlap in the age of amyloid positivity between DS and NT cohorts.
- Participants with DS accumulate amyloid 40% faster than those with sporadic AD.

## 1 INTRODUCTION

Down syndrome (DS) is characterized by the triplication of chromosome 21, which is implicated in multiple developmental and neurological sequelae for these individuals. People with DS have a lifetime estimated Alzheimer's disease (AD) incidence rate of 97%,^1^ and the average age of onset of dementia in the DS population is 54 years,^2^ with the accompanying presentation of AD symptoms over two decades earlier than neurotypical (NT) (i.e., non‐DS) populations.^3^, ^4^ This increased incidence of AD in DS is putatively driven by the overproduction of amyloid precursor protein (APP), which is regulated by a gene on the triplicated chromosome 21.^5^, ^6^, ^7^ A gene–dose effect is observed with this extra APP gene copy resulting in the generation of more amyloid beta (Aβ) in people with DS relative to their NT counterparts over their lifetime observed in *post mortem* data.^8^, ^9^

The earlier age of onset of Aβ and near‐certain prevalence of AD in the DS cohort offers a unique opportunity to identify and track the progression of AD biomarkers seen in preclinical AD and allows for the study of parallels between DS and sporadic AD. Using positron emission tomography (PET) imaging with carbon‐11 Pittsburgh compound B ([C‐11]PiB)^10^ compared to sporadic AD, earlier striatal deposition of amyloid is observed in DS preceding other areas of the cerebrum^11^, ^12^, ^13^ and is consistent with patterns seen in autosomal dominant AD.^14^, ^15^ The spatial distribution of cortical amyloid deposition outside the striatum, however, is consistent with sporadic AD, and biomarkers of tau deposition also follow similar spatial patterns of AD in NT populations.^16^, ^17^

Amyloid accumulation rate has previously been studied in sporadic AD with a focus on investigating time from amyloid deposition to other AD markers, including tau aggregation and cognitive impairment.^18^, ^19^, ^20^ In a recent investigation examining longitudinal modeling of amyloid deposition, Betthauser et al.^21^ found the rate of amyloid accumulation throughout the brain was relatively consistent between AD risk factors, sex, and apolipoprotein E (*APOE*) allele carrier status. Specifically, *APOE*4 carriers have a higher incidence of AD and accumulate amyloid at a younger age; however, the rate of amyloid accumulation was consistent with older adults who were *APOE*4 non‐carriers. Although people with DS similarly have a higher incidence rate of AD and demonstrate an earlier onset of AD‐related pathology, the rate of amyloid accumulation in the DS population and comparisons between this group and NT imaging cohorts have not been closely studied. In a previous study from our group, no significant difference in the rate of amyloid accumulation was found using the amyloid load model in DS participants^4^ compared to NT participants in the Alzheimer's Disease Neuroimaging Initiative (ADNI) cohort published by Whittington and Gunn.^22^ This work, however, included only a limited number of participants with elevated levels of amyloid burden, and there was an acknowledged large variance in the linear rate of change in global [C‐11]PiB SUVR used to derive the DS population amyloid accumulation rate.

With the known differences in amyloid production and age of onset of AD pathology in DS, the goal of this work is to more precisely evaluate the rate of accumulation of Aβ plaques between DS and NT sporadic AD populations imaged with [C‐11]PiB as they become Aβ+ followed over the course of many years. To maximize the precision of the measured progression of Aβ as participants become Aβ+ in this study, a small subset of our imaging cohort with longitudinal data covering the beginning of detectable amyloid deposition in the brain was identified. While this more focused group included fewer individuals compared to our previous analyses, the longitudinal design ensured our analysis was able to model individual participant amyloid trajectories over the course of presymptomatic AD for a more detailed characterization of variation seen at the individual level and make comparisons between populations at the same point in the AD timeline in order to inform future clinical trials using anti‐Aβ therapies.

## 2 METHODS

### 2.1 Imaging cohorts

Participants in this study were included from PET imaging studies at the University of Wisconsin–Madison and the University of Pittsburgh. DS participants were included from the Alzheimer Biomarker Consortium–Down Syndrome (ABC‐DS) study, an international, multicenter study that examines biospecimen, clinical, cognitive, and neuroimaging data to better understand the development of AD in the DS population. NT participants were selected from studies at both sites primarily focused on older adults enriched with individuals with familial risk of AD that included [C‐11]PiB PET imaging. Institutional Review Board approval and informed consent were obtained during enrollment into the studies by the participant or legally designated caregiver according to the Declaration of Helsinki. Inclusion criteria for participants in these analyses required having a minimum of three [C‐11]PiB scans with at least one Aβ+ (corresponding to a global SUVR > 1.40) and one Aβ− scan, that is, crossing the threshold for Aβ+. In total 20 DS participants and 23 NT participants at the two imaging sites fulfilled these requirements (Table 1), with several participants having over 10 years of [C‐11]PiB data and as many as seven individual scans.

**TABLE 1:** Description of Down syndrome (DS) and neurotypical (NT) carbon‐11 Pittsburgh compound B groups.

Parameter	DS	NT	*p* values
Participants (*n*)	20	23	–
PET scans (*n*)	78	109	–
PET scans per participant (*n*)	3.9 ± 0.6	4.7 ± 1.1	0.006
Time between scans (years)	3.0 ± 0.4	2.7 ± 1.0	0.29
Time from first to latest scan (years)	7.9 ± 1.9	9.4 ± 2.4	0.02
Female (%)	60%	61%	0.96
Age at first scan (years)	39.8 ± 5.9	72.2 ± 9.4	< 0.001
*APOE*4+ (*n*, %)	3, 15%	11, 48%	0.02
MCI at latest visit (*n*, %)	2, 10%	6, 26%	0.19
Dementia at latest visit (*n*, %)	3, 15%	5, 22%	0.58

Abbreviations: *APOE*, apolipoprotein E; MCI, mild cognitive impairment; PET, positron emission tomography.

Cognitive status for participants was determined using a consensus review committee as outlined in Handen et al.^23^ for participants with DS and in Johnson et al.^24^ for NT participants to identify cognitive decline consistent with mild cognitive impairment (MCI) and dementia in AD.

### 2.2 PET imaging

Participants in this study were imaged using [C‐11]PiB 50–70 min after injection with a nominal injected dose of 15 mCi. All images were acquired at the University of Wisconsin or the University of Pittsburgh using three different PET scanner types. A majority of participant scans (*n* = 152) were collected using the ECAT EXACT HR+ scanner model and reconstructed using the Direct Inverse Fourier Transform (DIFT) algorithm (image size:128 × 128 × 63, voxel size: 2.57 × 2.57 × 2.43 mm^3^). Additional images (*n* = 35) were taken using a Biograph Horizon PET/CT using iterative reconstruction (4i, 8sub) with an all‐pass filter (image size: 360 × 360 × 109, voxel size: 1.03 × 1.03 × 2.03 mm^3^) or a biograph PET/MR scanner reconstructed using filtered back projection with an all‐pass filter (image size: 256 × 256 × 109, voxel size: 1.03 × 1.03 × 2.03 mm^3^).

**RESEARCH IN CONTEXT**

1. **Systematic review**: Previous work demonstrated an earlier age of Aβ onset in DS compared to sporadic AD. A dedicated investigation into the rate of accumulation of amyloid in DS and a comparison to sporadic AD, though, has not been conducted.
2. **Interpretation**: Our findings reveal a faster rate of amyloid accumulation in the DS population compared to NT individuals without autosomal dominant AD. People with DS on average were estimated to accumulate amyloid 40% faster than their NT counterparts.
3. **Future directions**: Differences between individuals with DS and sporadic AD will be important considerations for future AD clinical trials and studies including the DS population. A wide range of amyloid accumulation rates were observed in the DS and NT imaging populations. Further work in this group will need to examine additional genetic and lifestyle factors to determine their influence on amyloid plaque accumulation.

### 2.3 Image processing

PET scans were processed using a standardized pipeline for comparison in a common space. First, individual PET frames were aligned to correct for interframe motion and then averaged from 50 to 70 min. The images were then spatially normalized into standard space using a [C‐11]PiB template created using the methods described in Lao et al.^25^ This previous work in the DS population demonstrated that the PET template provides normalization performance equivalent to that of a magnetic resonance imaging (MRI)‐based method, while also allowing for the inclusion of participants with low image quality MRI scans. Separate DS and NT templates were created using these methods for the two populations based on the differences in brain morphology seen in DS.^26^ These images were then converted into standardized uptake value ratio (SUVR) images using the cerebellar gray matter as a reference region as defined by the Automated Anatomical Labeling (AAL) atlas.^27^ To harmonize across different sites and scanners a three‐dimensional smoothing kernel of 6 mm^3^ was applied isotopically to the standard space SUVR images. The imaging processing methods followed previously established practices in AD studies in the DS population,^4^, ^17^, ^28^ with the same methods applied to the NT population for consistency for comparison. Global SUVR was calculated for all participants using a mask that was also generated from the AAL atlas described in Zammit et al.,^4^ including the anterior cingulate, frontal cortex, parietal cortex, precuneus, and temporal cortex. The striatum, an area demonstrating prominent Aβ burden in the DS population,^11^, ^29^ was excluded from this mask. An average global SUVR in these regions greater than 1.40 was considered Aβ+ corresponding to an amyloid load value of 20 or 33 Centiloids, as determined previously when defining amyloid load in the DS cohort by Zammit et al.^4^ However, it should be acknowledged that a lower threshold of 18 Centiloids was later reported based upon longitudinal analyses in the DS population.^28^ Global SUVR was used as a metric of amyloid accumulation (as opposed to amyloid load or Centiloids) to replicate the methodology of previous investigations in amyloid rate and because the amyloid load index incorporates the rate of amyloid accumulation in its parametric modeling.^4^, ^22^

### 2.4 Amyloid accumulation model

**FIGURE 1:**
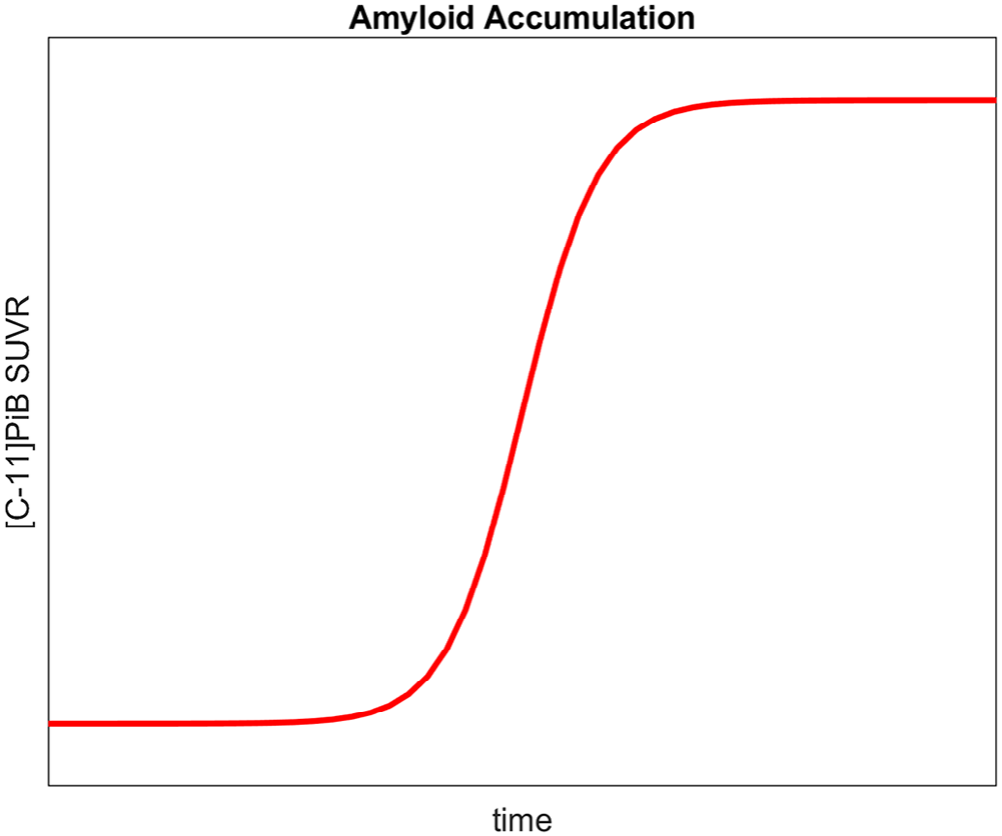
Expected trajectory of amyloid accumulation in brain over time as measured by positron emission tomography imaging.

Previous investigations into amyloid accumulation in the sporadic AD population proposed a logistic growth model to describe amyloid accumulation as a function of age using amyloid load,^22^, ^30^ which was subsequently validated in the DS population.^4^ Rather than assuming a linear trend, as amyloid burden begins to accumulate, it increases exponentially before reaching the amyloid carrying capacity (*K*) of the brain for amyloid as a function of increasing age (Figure 1). Although *K* is unique for different brain regions, taken together, the amount of Aβ deposited in individual brain regions can be combined into a global carrying capacity that models amyloid accumulation across the brain, which is used in these analyses. The time course of the amyloid burden in the brain measured by the PET scans is given by

(1) $$SUVR\;\left(t\right)=SUVR_{BL}+\frac{K}{1+e^{-r\left(t-t_{50}\right)}},$$

where *r* gives the steepness of the slope, which is used in this analysis as the outcome measure of the rate of amyloid accumulation. *SUVR_BL_* represents the baseline global SUVR for our population and was fixed to a value of 1.12 SUVR, which is the average global [C‐11]PiB SUVR of all amyloid‐negative participants imaged on the University of Wisconsin HR+ scanner. There was not a significant difference in baseline between NT (1.12 ± 0.07) and DS (1.10 ± 0.10) populations observed, and so *SUVR_BL_* was set to 1.12 for both populations. The carrying capacity (*K*) of amyloid in the cortices sets the upper limit of our model. This was set to 1.34, corresponding to a total SUVR of 2.46 (= 1.12 + 1.34), the highest global SUVR observed in either imaging cohort. Note that the participant who set the high global SUVR limit was not included in our logistic growth analysis due to not having any Aβ‐scans. Similar to the baseline global SUVR of our populations, the highest SUVR in the NT population (2.42) and DS populations (2.46), selected from the entire NT and DS cohorts, had similar values and were thus set to a single value for each population. The *t*_50_ term in the logistic growth model represents the time point at which the model reaches 50% of the difference between the upper limit and *SUVR_BL_*. The parameters *t*_50_ and *r* were previously shown to be consistent across different brain regions in NT^22^ and DS populations.^4^

Individual (i.e., participant) [C‐11]PiB Aβ trajectories were fit with the logistic growth model in MATLAB using the “fit” function with non‐linear least‐squares curve fitting. Comparisons between the estimated growth rates of the DS and NT cohorts were completed using a Student's *t*‐test with an α for significance set to 0.05. An average growth rate (*r*) was also estimated from each DS and NT combined group by fixing the *t*_50_ = 0 for each participant by normalizing the scan date for each participant to the individual estimates of *t*_50_. All time points in the DS and NT cohorts were modeled with one logistic growth curve respectively to give an estimate of the population growth rate and to calculate 95% confidence intervals.

The logistic growth model requires a plateau of the measured amyloid burden that is proportional to the carrying capacity (*K*). However, accurate estimation of *K* is limited due to the attrition of participants undergoing PET scans as AD‐related symptoms progress. As such, it is conceivable that the maximum observed [C‐11]PiB SUVR of 2.46 could yield an underestimated value of the true carrying capacity. To examine the implications of fixing *K* = 1.34, higher values of *K* were iteratively incorporated into the logistic growth model to evaluate if a bias was observed in the growth rate (*r*). Starting at *K* = 1.34 and moving in steps of 0.10, we increased the carrying capacity up to *K* = 2.94, corresponding to a SUVR = 4.06, for each cohort, re‐estimating the growth rate using the logistic growth model with the higher value for *K* at each step.

A minimum of three [C‐11]PiB scans was selected as an inclusion criterion for these analyses to fulfill the requirement of at least three data points for the estimation of two unknowns (*t*_50_ and *r*) in the logistic growth model. This restriction resulted in a reduced number of total participants compared to previous analyses.^4^ A secondary analysis was performed to include all participants with two or more scans using the Sampled Iterative Local Approximation (SILA) model previously reported by our group.^21^ Briefly, the SILA model provides an estimated time from biomarker onset (in this case Aβ+) by generating a continuous curve of expected biomarker accumulation based on longitudinal data. Participants with two or more [C‐11]PiB scans (regardless of Aβ positivity) were input into the SILA model, and curves were derived independently for the DS (*n* = 57) and NT (*n* = 162) populations. Then the SILA‐based estimated time from Aβ+ based upon the trajectory model was applied to all DS and NT [C‐11]PiB data from our site, including 239 NT and 199 DS participants for a total of 992 scans, normalizing them in time. The full DS and NT population data were then fit using the logistic growth model (similar to the method using scans normalized to *t*_50_ = 0 described previously) to derive population‐specific amyloid accumulation rates (*r*).

## 3 RESULTS

### 3.1 Global amyloid trajectories

A total of 43 participants were included in these analyses with 20 DS and 23 NT individuals meeting selection criteria. The NT group had a significantly higher number of scans per participant (*p* = .006) and time between first and latest [C‐11]PiB scan (*p* = .02). Age at time of first scan (*p* < .001) and proportion of *APOE*4 carriers (*p* = .02) were also higher among NT participants. No significant differences were observed based on cognitive status, sex, or time between scans. At baseline (i.e., the participant's first scan), NT participants included in this study had a higher global SUVR (1.28 ± 0.07) compared to DS participants (1.20 ± 0.08) (*p* = 0.002); however, in both cases baseline SUVR was elevated compared to the *SUVR_BL_* calculated for the logistic growth model from the full Aβ cohorts. There was no significant difference between global SUVR at the final scan for the NT (1.68 ± 0.15) and DS populations (1.64 ± 0.19) (*p* = .43).

**FIGURE 2:**
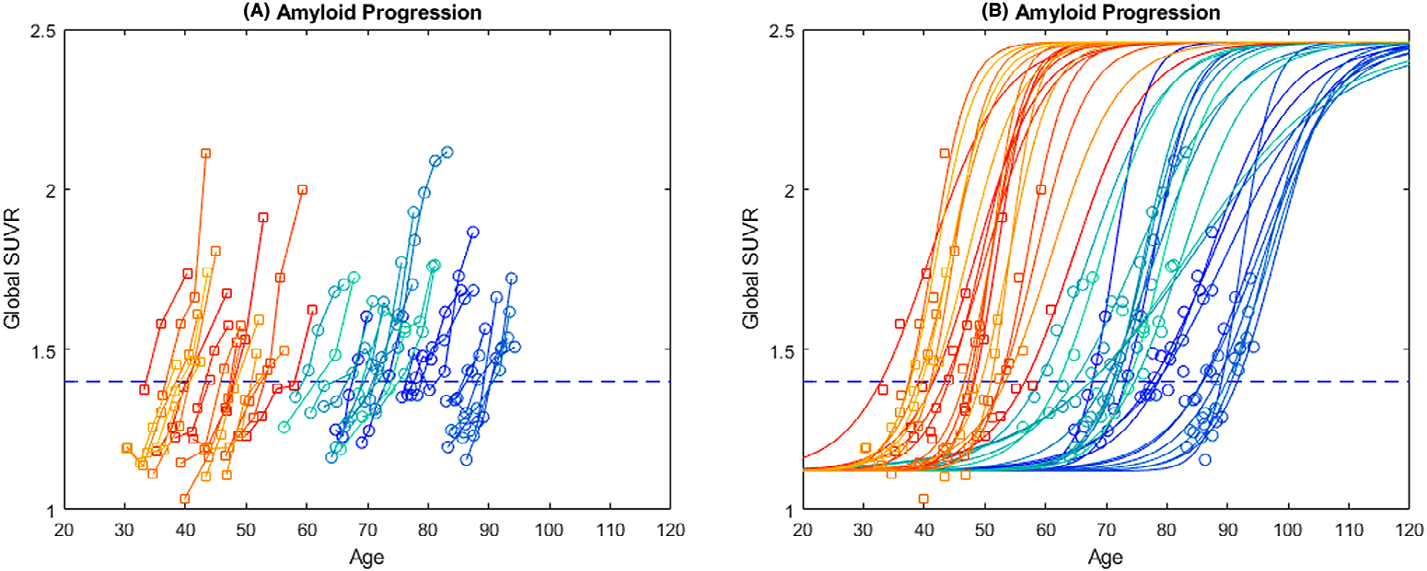
Amyloid trajectory plots for Down syndrome (warm colors) and neurotypical (cool colors) participants included in this analysis. Note that individual trajectories have different shades of warm or cool colors, so they are more easily distinguished from those of other participants. Individual participants are plotted piecewise linear (left) and using the logistic growth model (right) with the estimated *r*.

A clear delineation in age of onset was seen between amyloid positivity in the DS and NT populations. As shown in Figure 2A, there is no overlap between the trajectory plots of the two cohorts, with the latest DS participant becoming positive at age 56.6 years before the earliest NT participant became positive at age 58.9 years. Using the logistic growth curve, the estimated age of amyloid positivity for each participant was found to be significantly older in the NT group compared to the DS group (*p* < .001), 76.1 ± 9.6 years versus 45.2 ± 6.2 years, respectively.

**FIGURE 3:**
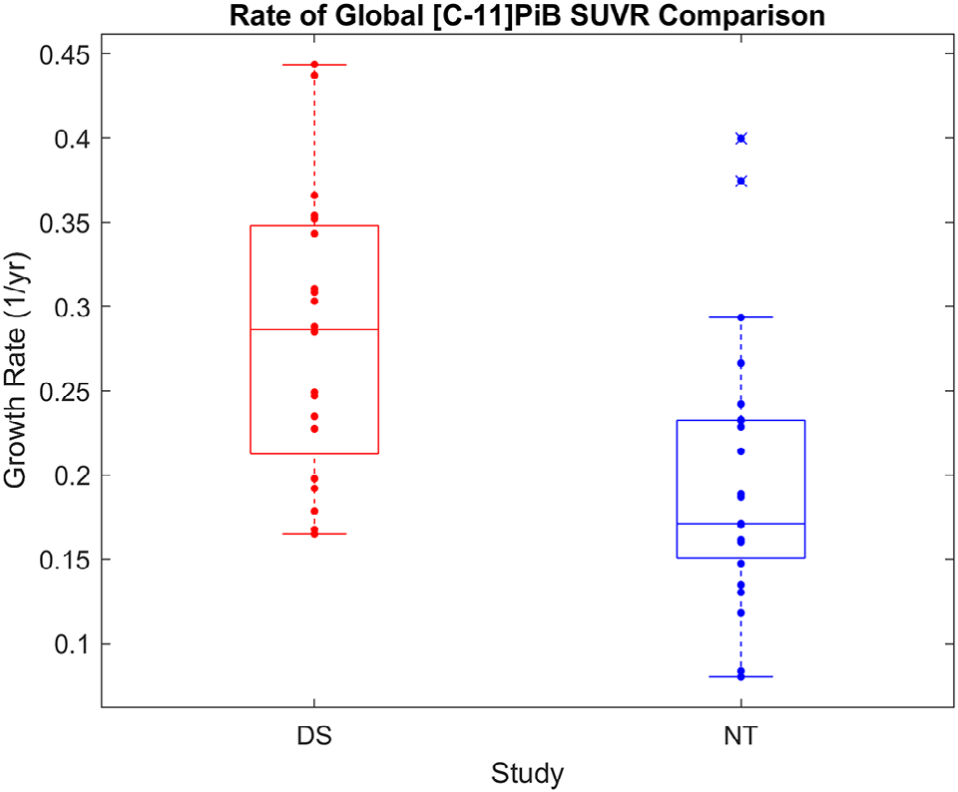
Box plots showing distribution of estimated growth rates represented by *r* in logistic growth model.

Shown in Figure 2B are the estimated functional equations of the measured [C‐11]PiB data, with each participant having between three and seven scans over the span of 4.0–13.3 years. The growth rate (*r*) calculated using our model revealed significant differences in the rate of PET‐measured Aβ plaque deposition between the DS and NT cohorts as they accumulated amyloid neuropathology. As shown in the box plots of Figure 3, the DS cohort reveals a growth rate of 0.28 (0.08)/year compared to 0.20 (0.08)/year in the NT group (*p* = .002). This increase of 40% corresponds to an annual change in SUVR (calculated using the derivative of Equation (1) with SUVR = 1.40) of 0.063 (0.018)/year and 0.044 (0.017)/year at the point of amyloid positivity. The estimated time from baseline (i.e., the last time point SUVR = 1.12) to Aβ positivity in the logistic growth model was also shorter for the DS population (13.7 ± 4.1 years) compared to NT (21.0 ± 8.7 years) (*p* = .001), potentially reflecting the compressed time frame of AD in DS from the faster accumulation of amyloid. A higher coefficient of variation ($\mathrm{COV}=\frac{\sigma }{\mu }$) is observed with the NT population (0.40) compared to DS (0.29), although this is primarily driven by the two outlier points, which can be seen over the NT box plot.

**FIGURE 4:**
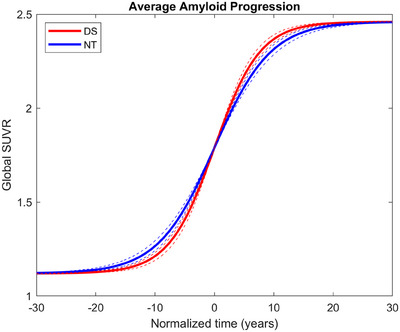
Average amyloid trajectories of Down syndrome and neurotypical populations measured using carbon‐11 Pittsburgh compound B normalized to *t*_50_ = 0.

Using the estimated *t*_50_ derived from the logistic growth model for each participant, the data were then normalized to the individual PET scans in time for comparison. Fitting all of the DS and NT scans with a single curve (Figure 4), the growth rate for the normalized DS is 0.26/year compared to 0.21/year in NT with 95% confidence intervals of [0.24, 0.29] and [0.19, 0.23], respectively. There is no overlap of the confidence intervals, further supporting the significant differences in [C‐11]PiB growth rate between these two populations.

**FIGURE 5:**
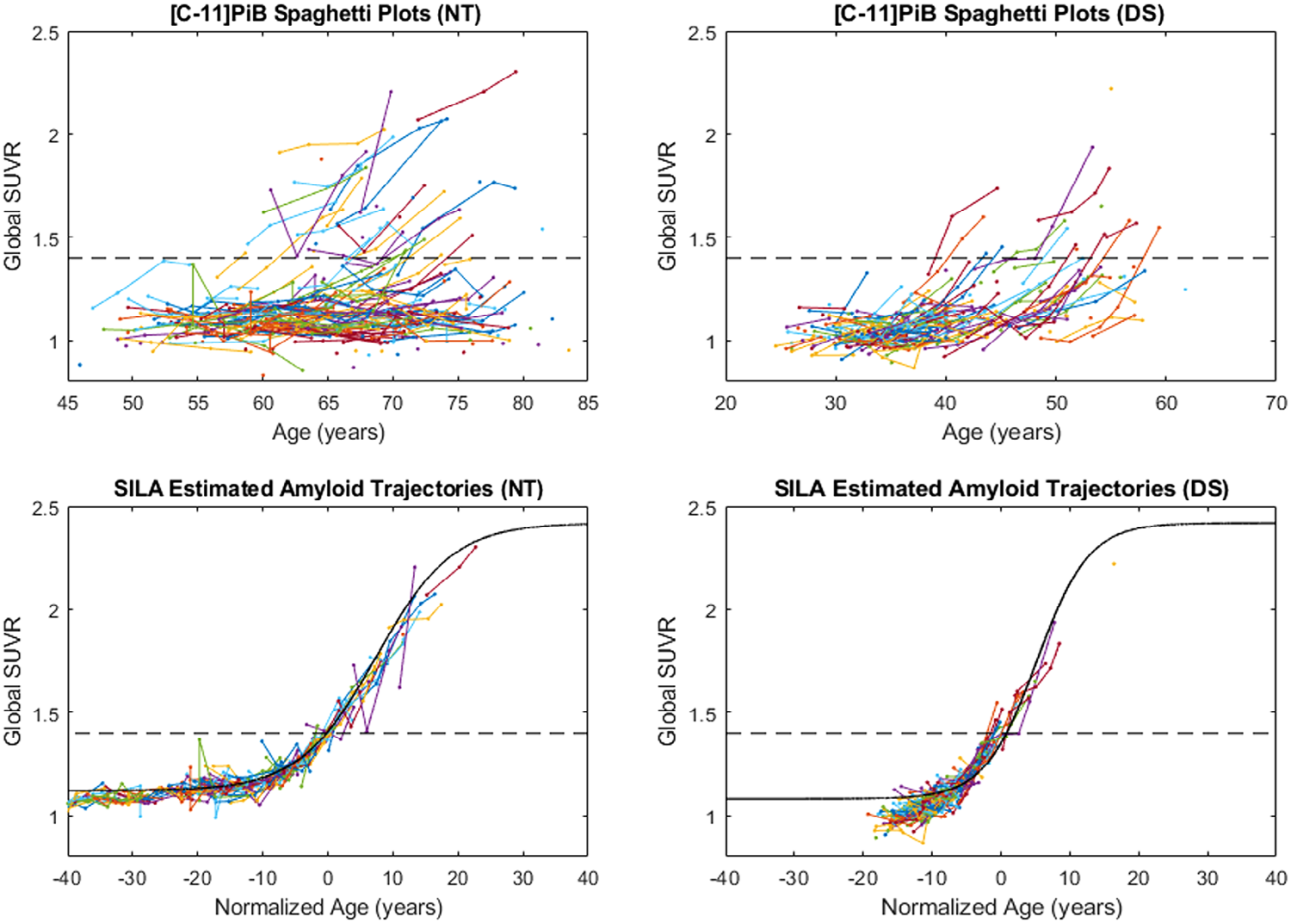
Trajectory plots for neurotypical (left) and Down syndrome (right) populations before (top) and after time normalization (bottom) using the Sampled Iterative Local Approximation (SILA) model. The SILA normalized data were then fit with the logistic growth model shown in this figure with the black line.

Using the SILA model to normalize the [C‐11]PiB trajectory plots in time (Figure 5), the estimated time from Aβ+ to *t*_50_ derived from the logistic growth model is decreased in DS (5.2 years) compared to NT (8.8 years). Similarly, the rate of accumulation is higher in DS with a value of 0.26/year compared to 0.17/year in NT with 95% confidence intervals of [0.15, 0.20] and [0.23, 0.29], respectively. The amyloid accumulation rates derived using the logistic growth model in the primary analyses with at least three scans are within the 95% confidence intervals as well

### 3.2 Effects of carrying capacity

**FIGURE 6:**
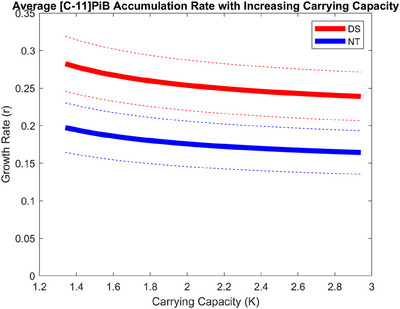
The growth rate of the Down syndrome and neurotypical cohorts with a higher carrying capacity used in the logistic growth model, with 95% confidence intervals represented by the dashed lines and with an increasing upper limit on the model the growth rate decreased for both populations, but at a similar rate, so there was not an overlap between confidence intervals for any of the carrying capacity values observed.

To examine the assumption of setting the carrying capacity to a fixed value, this term was iteratively increased to test if the higher asymptotic value would influence the estimated growth rate (*r*). In both groups increasing the upper limit decreased the estimated growth rate until eventually leveling off to values of around *r* = 0.15/year and 0.22/year for NT and DS populations, respectively (Figure 6). The *p* values comparing the two cohorts were never greater than .002 for any value of the observed carrying capacities.

## 4 DISCUSSION

PET imaging studies observing amyloid burden have consistently demonstrated that Aβ plaques are present at a younger age in individuals with DS compared to NT (non‐autosomal dominant) populations. The results from this study replicated these findings, with an average difference in age of amyloid plaque onset of 31.1 years observed between DS and NT groups. Our findings support the finding that people with DS also have a compressed time frame in AD neuropathology compared to sporadic AD in the NT population.^17^ In light of promising new anti‐amyloid therapies, there is great interest in including people with DS in clinical AD trials both among researchers and members of the DS community. The effectiveness of anti‐amyloid therapies is quantified by changes in amyloid burden measured by amyloid PET imaging, and differences in amyloid accumulation could influence the treatment design for clinical trials involving people with DS and understanding of the relationship between amyloid burden and decline in cognitive function.^31^

In the original work applying the amyloid load index to the DS population, no differences were found in the rate of accumulation between NT and DS populations.^4^ While a comparison of amyloid accumulation rate was not the goal of that earlier study, a logistic growth rate of *r* = 0.21/year was calculated for the DS population using an integrated restricted spline method compared to *r* = 0.20/year from the ADNI cohort.^22^ A central aim of the work presented here was to carefully re‐examine the growth rate given additional available longitudinal data and to more precisely model the rates of Aβ accumulation due to some known limitations (e.g., high variability) of the previous study. In the previous comparison between DS and ADNI cohorts, different PET radiotracers and image processing methods were used. To minimize any variance in methodology, the current study restricted individuals to a single PET radiotracer ([C‐11]PiB) and included only two sites with large ongoing longitudinal studies of Aβ PET in both NT and DS cohorts. Additionally, a nearly identical processing pipeline was used for both cohorts, with the only difference being the PET normalization templates used for NT and DS. Although the methods were modified from the original publications, we were still able to replicate the NT growth rate of *r* = 0.20/year found in the ADNI cohort with the included NT subjects.

There was also an acknowledgment of the low number of participants with elevated [C‐11]PiB SUVR (i.e. Aβ+) included in the original work. This limited the ability to accurately model amyloid deposition near the upper limit of [C‐11]PiB global SUVR. While the current study also included a small number of high global SUVR PET images, a careful examination of the implications of using an a priori upper limit was conducted using a range of carrying capacity values in the logistic growth model (*K*). At higher values of *K*, there continued to be significant rate differences between cohorts beyond the maximum global [C‐11]PiB SUVR observed in the DS imaging cohort or reported in the literature. Due to known morphological differences between DS and NT populations,^32^ it is acknowledged there may be different carrying capacities for amyloid between these two populations. However, we have not observed significant differences in our imaging populations. Maintaining the NT carrying capacity value of 1.34 while varying the DS carrying capacity demonstrates the difference in growth rate remains significantly higher for DS participants up to a value of *K* = 2.42, well above any global SUVR values observed at any of the ABC‐DS imaging sites.

The implementation of the logistic growth model resulted in a reduced sample size when considering the overall total numbers of PET scans performed in both the DS and NT cohorts. Careful selection of participants allowed for the analysis to focus its attention on individuals who were longitudinally accumulating amyloid for direct comparison between populations while limiting sources of variance observed in previous investigations. This study primarily focused on the longitudinal rate of amyloid accumulation in DS, and although over 200 participants were imaged using [C‐11]PiB in the ABC‐DS study, only 35 had longitudinal data and an Aβ+ scan (i.e., are actively accumulating amyloid). Results incorporating the full [C‐11]PiB imaging cohort with the SILA model saw close agreement to the amyloid accumulation rates seen with our more focused analysis.

Another investigation from our group also examined amyloid accumulation rate between DS participants and non‐demented elderly (NDE) NT participants.^33^ This investigation reported significantly faster Aβ accumulation in the anterior ventral striatum for DS participants, but a slower rate in the frontal cortex and precuneus. Note that this earlier work included only cognitively stable participants, while in the analysis here, 48% of NT participants and 25% of DS participants were cognitively impaired and on average were further along in AD progression than participants in the previous study. Given the early striatal amyloid deposition in the DS population, which occurs >5 years before cognitive impairment, the Tudorascu et al.^33^ study was particularly sensitive to the difference in early accumulation between DS versus NT populations. However, the logistic growth model used in this study was more sensitive to the differences in changes in amyloid after the onset of amyloid positivity.

In the scientific literature, there is a range reported in the appropriate threshold for Aβ+.^34^ In this study, a relatively high value of a global SUVR of 1.40 corresponding to 33 Centiloids was used. The threshold for Aβ+ was used as a selection criterion, so participants were normalized within a consistent time frame of amyloid accumulation. Setting a lower threshold might have changed the makeup of the cohorts, as some participants would not have had a global SUVR bracketing this new cutoff value. We do not anticipate this would alter our estimated rate of accumulation, although it would shift the estimated age of amyloid positivity to an earlier time.

A few demographic differences between the NT and DS cohorts must also be acknowledged. Due to the established earlier age of onset of AD and amyloid deposition, the DS participants included in this study were on average over 30 years younger than their NT counterparts. Past work in amyloid accumulation found closer agreement between the duration of amyloid positivity and the trajectory of amyloid accumulation compared to the relationship between amyloid and age at the time of baseline scan.^35^ In limiting our search to participants that converted from amyloid negativity to positivity during the studied period, we selected participants that were normalized in time to include the onset of amyloid accumulation despite differences in age of onset between populations. This method mirrors previous work in the DS population,^17^ and no further corrections for age were included in these analyses.

NT participants were included from studies at the Universities of Wisconsin and Pittsburgh studying older adults, enriched with participants with familial history and risk factors for AD. The NT studies with [C‐11]PiB have been ongoing for several years longer than the DS‐related studies (since 2003 and 2010 respectively), which explains the significant differences seen in the number of scans and duration. A higher percentage of *APOE*4 carriers is present in the NT group (48%) than in the DS cohort (15%); however, previous work in the sporadic AD population showed a consistent rate of amyloid accumulation among different AD risk factors, including sex and *APOE*4 carriage.^21^ While the age of onset is earlier for *APOE*4 carriers, the accumulation rate did not significantly differ, and it is unlikely that the demographic differences biased the results presented in this study.

Harmonization between sites and scanners involved smoothing with a 6‐mm^3^ isotropic kernel. This method follows previous work in the DS population, including the original investigation of amyloid accumulation rate in DS,^4^ which used similar methods as the ADNI cohort.^22^ Scanner effects both within and between sites were examined in this investigation, and a more thorough discussion of the harmonization considerations for this project is included in the supplementary material.

While on a population level there was a separation between the estimated growth rates of the DS and NT populations putatively driven by the gene–dose effect accompanying the extra copy of the APP gene, the differences in age of onset and growth rate between these groups may involve additional factors beyond trisomy 21. The difference in age of Aβ positivity between the oldest DS participant and youngest NT participant included in the analysis was less than 3 years, and the range of logistic growth rates observed had a large overlap, covering [0.08–0.40/year] and [0.17–0.44/year] for the NT and DS cohorts, respectively. Further work in the ABC‐DS study will examine the effect of other genetic and lifestyle factors, as well as other AD biomarkers, on amyloid accumulation at both population and individual levels beyond APP expression. Moving forward, establishing the differences in how amyloid accumulates in the brain for participants with DS versus without DS is critical information for clinical trials looking at amyloid clearance with the DS population and beyond.

## CONFLICT OF INTEREST STATEMENT

The authors have no conflicts of interest to disclose. Author disclosures are available in the Supporting Information.

## ETHICS STATEMENT

Institutional Review Board approval and informed consent were obtained during enrollment in the studies by the participant or legally designated caregiver according to the Declaration of Helsinki.

## Supporting information



Supporting Information

Supporting Information
