# Supplementary material for: PET‐measured amyloid beta accumulates at an accelerated rate in Down syndrome compared to neurotypical populations
Source: Alzheimers Dement. 2025 Jun 24;21(6):e70357. doi: 10.1002/alz.70357 (PMC12187968; doi:10.1002/alz.70357)
Supplement: Supplementary file 1 — Supporting Information [file ALZ-21-e70357-s002.docx]

**Supplemental Material:**

The inclusion of only two sites in these analyses was motivated by the goal to reduce across-site variation in derived imaging outcomes due to differences in experimental acquisition methods, including PET scanner model and reconstruction algorithms. However, we note that both sites included upgraded PET scanner equipment over the 10+ years spanning these studies, and many participants transitioned between scanners between visits. A uniform processing pipeline was applied to all imaging data and smoothing was performed as the final step using a 6mm isotropic smoothing kernel to harmonize across scanners, however, recent research suggests that smoothing alone may be insufficient in removing biases due to scanner differences [1]. While the comparison of PiB growth rate is downstream from the global SUVR measure, whose values would directly reflect any scanner biases, many of the participants included in this analysis were imaged on multiple scanners. Harmonization methods such as the ComBat algorithm [2] were found to be a poor fit for this analysis, though, due to a large mismatch in age and disease progression between scanners, particularly at the University of Wisconsin. Many of the DS participants at the University of Wisconsin were initially scanned on a Siemens ECAT HR+ scanner under a previous protocol before transitioning to a Horizon Biograph mCT for their most recent timepoints, and on average participants on the University of Wisconsin Biograph were older and had a higher amyloid burden than those acquired on the HR+. Harmonization methods between scanners normalized both scanner effects and the increase in amyloid burden observed with the participants who were imaged on both systems. In order to explore any potential scanner bias to the results without global SUVR correction, the growth rates of participants with three or more scans on a single scanner were compared with the full imaging population (including those imaged on multiple scanners) both with and without smoothing.

For participants imaged only on one scanner and with a 6mm isotropic smoothing kernel, the average growth rate for DS and NT groups was 0.26(0.06)/year and 0.20(0.08)/year respectively (p = 0.03). Without smoothing, the growth rate averages were 0.28(0.08) and 0.20(0.06) (p < 0.001) for all participants and 0.26(0.06) and 0.20(0.06) (p = 0.01) for participants imaged on a single scanner. Ultimately, the results were consistent between methods and scanners and the results remained significant with and without smoothing. In order to maximize the number of scans and participants, the inclusion criteria did not limit the presented analysis to just participants imaged on a single scanner. These results demonstrate that scanner effects did not drive the observed differences between DS and NT cohorts reported in this study.

**References:**

[1] Torbati ME, Minhas DS, Laymon CM, et al. MISPEL: A supervised deep learning harmonization method for multi-scanner neuroimaging data. *Med Image Anal*. Oct 2023;89:102926. doi:10.1016/j.media.2023.102926

[2] Beer JC, Tustison NJ, Cook PA, et al. Longitudinal ComBat: A method for harmonizing longitudinal multi-scanner imaging data. *Neuroimage*. Oct 15 2020;220:117129. doi:10.1016/j.neuroimage.2020.117129
